# Editorial for the Special Issue on ‘Metal Oxide Thin Film: Synthesis, Characterization, and Application’

**DOI:** 10.3390/ma14081834

**Published:** 2021-04-07

**Authors:** Erwan Rauwel, Protima Rauwel

**Affiliations:** Institute of Technology, Estonian University of Life Sciences, Kreutzwaldi 56/1, 51014 Tartu, Estonia; protima.rauwel@emu.ee

The last two decades have witnessed the development of new technologies for thin-film deposition and coating. A marked improvement in thin-film deposition methods, enabling the precise coating of surfaces at the nanoscale on complex nanostructure and on large surfaces is viable. These include new deposition methods like atomic layer deposition (ALD) that enables the coating on complex nanostructures with high conformality and homogeneity. Other more conventional methods based on coating in solution have also been improved in order to achieve a high quality conformal coating. These deposition processes also enable the synthesis of multilayers and the development of synergistic effects through the mutual interaction of such layers. At the same time, a need for new tools to characterize these films has emerged. This Special Issue is dedicated to these recent developments and contains 10 manuscripts reporting thin-film deposition and coating and their characterization. It compiles works on vapor-phase deposition processes including atomic-layer deposition (ALD) [1,2], mist-chemical-vapor deposition [3] and atmospheric-pressure chemical-vapor deposition (APCVD) [4] methods in which authors have studied the stabilization of metastable phases and the effect of growth conditions on thin film quality, epitaxy, and crystallinity. The Special Issue also includes liquid-phase coatings like sol-gel [5], solution immersion [6], micro-arc oxidation [7], Plasma Electrolytic Oxidation Processing [8], and single pulse anodization [9] where the authors were more focused on the coating of aluminum. The last paper reports on a new metal oxide thin film characterization method [10] that can extract the majority charge carriers’ type, their concentration, and mobility and is extendable to the study of other wide-bandgap semiconductors. Two of these manuscript reports studies are on Transparent Conductive Oxide thin films [4,10] and in one manuscript, the authors investigate InGaZnOx Thin Film Transistors [6].

The first four manuscripts of this Special Issue consist of thin-film depositions in gas phase or solution. The stabilization of NiTiO_3_ metastable phase using atomic layer deposition (ALD) was studied by Bratvold et al., in the manuscript “Phase and Orientation Control of NiTiO_3_ Thin Films” [1]. They demonstrated that it is possible to stabilize NiTiO_3_ disordered phase (R−3c) on α-Al_2_O_3_ substrates. They also investigated the influence of substrate on the symmetry and orientation of NiTiO_3_ thin films under epitaxial growth conditions on LaAlO_3_(100), SrTiO_3_(100) and MgO(100) substrates. They have demonstrated that the orientation and symmetry of NiTiO_3_ thin films deposited by ALD can be controlled by the choice of a suitable substrate. Using ALD, Duparc et al., studied the deposition of GdCoO_3_ and Gd_0.9_Ca_0.1_CoO_3_ thin films on LaAlO_3_(100) and YAlO_3_(100/010) substrates in “Atomic Layer Deposition of GdCoO_3_ and Gd_0.9_Ca_0.1_CoO_3_” [2]. These coatings may find applications in the field of oxidation catalysis. They showed that crystal orientations of the films can be tuned by the selection of the substrate and mitigated through the interface via solid-face epitaxy upon annealing. In the third manuscript, Xu et al. report on the influence of carrier gases in mist chemical vapor deposition method on the quality of epitaxial corundum-structured α-Ga_2_O_3_ films [3]. The thin films were grown on c-plane sapphire substrates with single (0006) plane orientation for all carrier gases, but the authors demonstrated that the O_2_ gas enables the growth of better quality epitaxial films with increased growth rate (10.3 nm/min vs. 5.3 nm/min and 2.4 nm/min) and better crystalline quality. The fourth manuscript reports the study of the influence of La doping in BiFeO_3_ thin films grown on Pt/TiO_2_/SiO_2_/Si substrates by the sol-gel method [5]. The authors have shown that it is possible to increase the grain size and self-polarization and promote (102) texture of the film by La doping and a higher growth temperature. These characteristic are important for future piezoelectric applications. They show that piezoelectric properties are connected to the films’ growth conditions and La doping, emphasizing that if both are thoroughly controlled BiFeO_3_ -based films could be used in micromechanical applications.

Manuscripts reporting on thin films grown by APCVD and solution immersion follow these studies. In “Origin of Magnetotransport Properties in APCVD Deposited Tin Oxide Thin Films” by Juraić et al., the structural and magnetotransport studies of tin oxide thin films prepared by the cost-effective Atmospheric Pressure Chemical Vapor Deposition method deposited on soda-lime glass substrates [4]. In particular, they studied the charge carrier density and mobility of the films that are typical values of SnO_2_ films manifesting a temperature dependence in their case. This behavior is attributed to grain boundary scattering and crystallites preferred orientation. In the second paper, “Optimizing the Properties of InGaZnOx Thin Film Transistors by Adjusting the Adsorbed Degree of Cs^+^ Ions”, Zhang et al. investigated surface structure modifications and increased oxygen vacancy concentrations of a-IGZO thin films through the introduction of Cs^+^ ions into the surface [6]. They observed an improvement of transfer properties and stability of the films coated by solution immersion method at low temperature. They found that the optimized performance of Cs-doped InGaZnOx Thin Film Transistors was obtained for a Cs^+^ ion concentration of 2% mol/L, with thin films that exhibit a carrier mobility of 18.7 cm^2^·V^−1^·s^−1^, OFF current of 0.8 × 10^−10^ A and a threshold voltage of 0.2 V.

The last set of manuscripts focus more particularly on aluminum coating using liquid phase coating. In these investigations, the authors mainly investigated how the coating methods could be improved and identified the main parameters that control the growth quality. Song et al. studied the tribological performances of micro-arc oxidation of aluminum 6061 alloy with the addition of 0.75 g/L of cellulose into the electrolyte [7]. They observed an improvement of the coating with a decrease of the roughness along with an increase of cellulose concentration in the electrolyte. They indicate that cellulose fills the micro-cracks and micro-pores and a part of cellulose is cross-linked with the Al^3+^. This study is followed by the report of Yang et al., in which the authors investigated a novel self-adaptive control method for large scale coating of aluminum alloys using plasma electrolytic oxidation processing [8]. They showed that coating can be controlled via feature information (Vb, Ib, Vi, and Vu) and the growth process can be tuned via the voltage, current, and processing time parameters. The coating exhibited good uniformity and compactness without visible cracks and large pores. For optimal growth process conditions on 6061 aluminum alloy samples, they measured a specific energy consumption of 1.8 kWh·m^−2·^μm^−1^. The same group also studied the discharge behavior and dielectric breakdown of oxide films deposited on aluminum in an alkaline silicate electrolyte using single-pulse anodization of aluminum micro-electrodes [9]. They measured voltage and current waveforms of applied pulses and studied the morphology of the coating. They found a good correlation between the pulse parameters and shape of discharge channels and showed that by increasing potential and pulse width it is possible to form circular opened pores on the surface.

The last paper entitled “Hot-Probe Characterization of Transparent Conductive Thin Films” describes the dynamic hot-probe measurement system that replaces or supplements existing methods like Hall effect measurements or the Haynes–Shockley experiments [10]. The replacement of indium–tin oxide by more cost-effective materials like ZnO doped by Al, Mn, or Sb requires mobile and low-cost evaluation methods. The paper shows that Dynamic Hot-Probe Characterization can extract the main parameters of metal oxide thin films like the majority charge carriers type, their concentration, and mobility and this method may also be applied to other wide-bandgap semiconductors.

We hope that this special issue will serve as a valuable support for researchers in the field of metal oxide thin-film deposition and characterization and we wish you a pleasant read.

## Data Availability

Not applicable.
